# Early and Long-Term Effects of Abdominal Fat Reduction Using Ultrasound and Radiofrequency Treatments

**DOI:** 10.3390/nu14173498

**Published:** 2022-08-25

**Authors:** Magdalena Kiedrowicz, Ewa Duchnik, Jolanta Wesołowska, Beata Bania, Małgorzata Peregud-Pogorzelska, Dominika Maciejewska-Markiewicz, Ewa Stachowska, Joanna Kruk, Mariola Marchlewicz

**Affiliations:** 1 Department of Dermatology and Venereology, Pomeranian Medical University in Szczecin, Police, ul. Siedlecka 2, 70-010 Police, Poland; 2 Department of Aesthetic Dermatology, Pomeranian Medical University in Szczecin, ul Powstańców Wielkopolskich 72, 70-111 Szczecin, Poland; 3 Cardiology Clinic, Pomeranian Medical University in Szczecin, ul Powstańców Wielkopolskich 72, 70-111 Szczecin, Poland; 4 Departament of Human Nutrition and Metabolomics, Pomeranian Medical University in Szczecin, ul. Broniewskiego 24, 70-240 Szczecin, Poland; 5 Faculty of Physical Culture and Health, Szczecin, University of Szczecin, Piastów 40b/6, 71-065 Szczecin, Poland

**Keywords:** abdominal fat reduction, ultrasound treatment, radiofrequency treatment, fatty acids

## Abstract

Background: Abdominal obesity, together with atherogenic dyslipidemia, increased blood pressure and impaired glucose tolerance, was included in the components of metabolic syndrome identifying patients at high risk of cardiovascular diseases. Subcutaneous adipose tissue is a buffer for dietary fatty acids (FAs). It was reported that the fatty acid composition of adipose tissue reflects the dietary intake of FAs in the previous 6 to 9 months. Therefore, we decided to evaluate the early and long-term metabolic effects of lipocavitation and/or thermolipolysis on abdominal fat reduction. Material and Methods: The study population comprised 60 women. Subjects were randomly allocated into one of three subgroups, 20 women per subgroup, and each subgroup received 10 treatments with ultrasound (U group), radiofrequency (RF group), or combined radiofrequency and ultrasound (RF/U group) for the abdominal region. Treatments were provided three times a week using the multifunctional device (Professional Beauty Equipment, HEBE, Warsaw, Poland). Each treatment to reduce adipose tissue, regardless of the method used, involved 20 min of massage with the dedicated applicator head on a rectangular 20 cm × 10 cm area of the abdominal region. Fatty acid composition and biochemical and anthropometric parameters were measured before the first, after 10 treatments and 6 months after the last treatment. Results and discussion: The series of 10 treatments to reduce abdominal adipose tissue using ultrasound, radiofrequency or both methods resulted in a cosmetic effect which was reflected in weight loss and BMI reduction. Reduced waist circumference was also found in patients who received radiofrequency or two types of intervention (RF + U) but not ultrasound treatments. The long-term cosmetic effect (lasting for at least 6 months) was achieved only with RF treatment and was reflected in reduced body weight, BMI and waist circumference. None of the treatments had a direct, short or long-term effect on the lipid profile, insulin resistance markers, inflammation markers, or blood pressure. Consequently, did not modify the risk of cardiovascular diseases.

## 1. Introduction

Abdominal obesity is a recognised, significant factor increasing the risk of cardiovascular diseases, both through the development of major classical risk factors and through other mechanisms, including dyslipidaemia or insulin resistance, eventually leading to atherosclerosis [1]. Therefore, abdominal obesity, along with atherogenic dyslipidaemia, increased blood pressure and impaired glucose tolerance, was included in the components of metabolic syndrome identifying patients at high risk of cardiovascular diseases [2].

Subcutaneous adipose tissue acts as a buffer for dietary fatty acids (FAs), which are stored and released under the influence of many different factors. It was reported that the fatty acid composition of adipose tissue reflects the dietary intake of FAs in the previous 6 to 9 months [3]. Recently, it was also demonstrated that the composition of FAs in the blood, although largely dependent on the diet, may correlate with FAs in adipose tissue [4]. The ratio of the consumed FAs, and in particular, the content of mono- and polyunsaturated FAs to saturated FAs in the diet, is important in the development of atherosclerosis [1]. 

Recent years have increased the popularity of aesthetic medicine treatments [5,6,7,8,9,10]. However, interventions such as liposuction, injection lipolysis, ultrasound, or radiofrequency treatments to reduce adipose tissue may create a risk of metabolic disorders with difficult to assess effects. During these procedures, adipose tissue lysis occurs due to damage to adipocyte cell membranes. It is assumed that triglycerides released from damaged adipocytes may be dispersed in the interstitial fluid. They are gradually transported through the lymphatic and blood systems to the liver [4].

Ultrasound used for body sculpting can be divided into two categories: relatively low-intensity/low-frequency nonthermal ultrasound and high-intensity focused ultrasound (HIFU). Ultrasound can lyse adipocytes through mechanical and thermal mechanisms. With nonthermal ultrasound, a low frequency is chosen to increase the likelihood of cavitation while generating little heat through absorption mechanisms. At lower frequencies, ultrasound can easily cause cavitation, creating holes (cavities) when the ultrasound wave has sufficient negative pressure to overcome the adhesion of the medium molecules to each other, causing cell death [9,11]. Low-intensity nonthermal (mechanical) ultrasound is approved for circumference reduction of the waist, hips, and thighs [9,11]. The mechanical energy may also be absorbed by the tissues and converted into heat, which can cause changes such as improved microcirculation, accelerated metabolism, activation of enzymatic reactions, increased elasticity of collagen fibres and increased permeability of cell membranes [12,13,14]. 

Used for body sculpting, HIFU delivers focused, high-intensity ultrasonic energy to deep subcutaneous tissue, producing heat capable of ablating adipose tissue and thermally modifying collagen [9]. Exposure of adipocytes to a temperature of 56 °C or higher for 1 s is adequate to cause rapid cell death by coagulative necrosis [8,9,11].

Another non-invasive method, radiofrequency, generates heat in different tissues by transforming energy through three basic mechanisms from an electromagnetic field. These mechanisms include (i) orientation of electric dipoles that already exist in the atoms and molecules in the tissue; (ii) polarisation of atoms and molecules to produce dipole moments; and (iii) displacement of conduction electrons and ions in the tissue [8]. Radiofrequency works primarily through skin tightening rather than destruction of adipose tissues, making it better suited for patients with cellulite than for reduction in waist circumference [8,9,11]. With monopolar radiofrequency devices, energy is passed from a single electrode into the skin and subcutaneous tissues and directed to a return pad in another area of the body, usually the back. With multipolar radiofrequency, two or more electrodes contained within the same handpiece are positioned at different points on the skin so that the waves pass between them to create heating. This latter method directs the trajectory of the current but not the depth [9,11]. The temperature of tissues increases to 45–55 °C. A large portion of the heat generated by radiofrequency electromagnetic waves is absorbed, for example, by subcutaneous adipose tissue, muscles, body fluids and parenchymal organs. Increased temperature causes vasodilatation and enhancement of tissue metabolism, as well as the destruction of the cross-links between collagen fibres. Skin and subcutaneous tissues are heated to the same extent. Radiofrequency emitting devices generally use skin cooling to protect the epidermis from thermal damage [9]. RF waves can be delivered in a continuous or pulse mode. In pulse mode therapy, the temperature increase is lower, but deeper penetration is achieved [15,16]. 

There are no numerous reports in the literature presenting the effects of non-invasive techniques for adipose tissue reduction on metabolic parameters significantly contributing to the pathogenesis of atherosclerosis. Therefore, we decided to evaluate the early and long-term metabolic effects of lipocavitation and/or thermolipolysis on abdominal fat reduction. 

## 2. Material and Methods

### 2.1. Study Population

**Figure 1:**
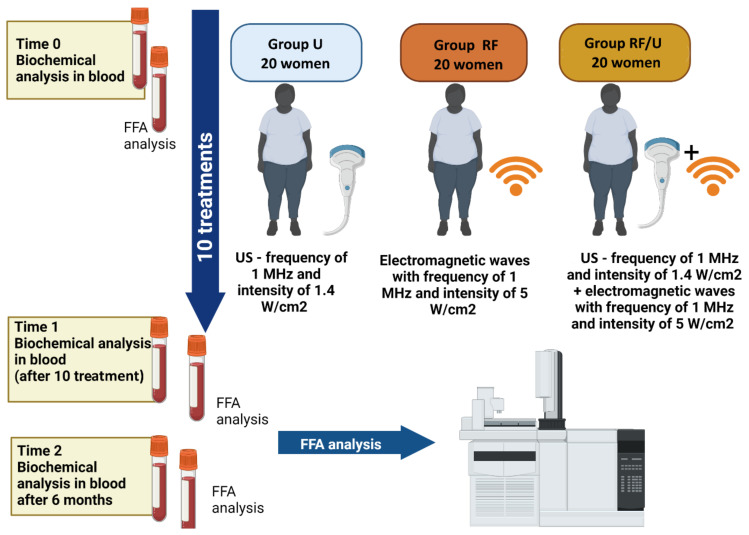
Study design (prepared using BioRender software).

The study population comprised 60 women with low risk of cardiovascular diseases assessed based on the guidelines of the European Society of Cardiology [1], but with excessive accumulation of adipose tissue in the abdominal region measured by bioimpedance analysis (BIA). The inclusion criterion was content of abdominal fat higher than 29% (TANITA AB-140 ViScan, Tokyo, Japan). Subjects were randomly allocated into one of 3 subgroups, 20 women per subgroup, and each subgroup received 10 treatments with ultrasound (U group), radiofrequency (RF group), or combined radiofrequency and ultrasound (RF/U group) for the abdominal region. Treatments were provided three times a week at the Department of Aesthetic Dermatology using a multifunctional device (Professional Beauty Equipment, HEBE, Wrocław, Poland). Patients with active infections and a history of autoimmune diseases were excluded from the study. Each patient gave written informed consent to participate in the study. Each treatment to reduce adipose tissue, regardless of the method used, involved 20 min of massage with the dedicated applicator head on a rectangular 20 cm × 10 cm area of the abdominal region. Ultrasound treatments were performed using an applicator head with a 38.47 cm^2^ spot area, generating ultrasound with 1 MHz frequency and 1.4 W/cm^2^ intensity [4]. Radiofrequency treatments were performed using an applicator with a 20 cm^2^ spot area generating electromagnetic waves with 1 MHz frequency and 5 W/cm^2^ intensity [4]. In the RF/U group, massage with a radiofrequency applicator head (20 min) was followed by a massage with a head generating ultrasound (20 min). During the study, participants continued their usual diet and physical activity. Study design was shown in Figure 1.

### 2.2. Anthropometric Measurements

Standard medical tests of patients were conducted at the Department of Cardiology with the Intensive Cardiac Care Unit. The following anthropometric parameters were measured in each of the patients: body height (in cm), body weight (in kg), waist and hip circumference (in cm), abdominal fat content, visceral fat content (in%), and blood pressure. Body weight and height were measured with a certified device (Radwag, Szczecin, Poland). The waist and hips circumferences were measured with a medical tape measure, and the content of abdominal fat and visceral fat was determined by bioimpedance analysis (TANITA AB-140 ViScan, Japan) at the Department of Aesthetic Dermatology. The measured parameters were used to calculate the body mass index (BMI) and the waist-to-hip ratio (WHR) [1]. 

### 2.3. Biochemical Parameters

Patients from all study subgroups were tested for white blood cell (WBC) count in peripheral blood and levels of glucose, insulin, C-reactive protein (CRP), total cholesterol (TC), LDL cholesterol, HDL cholesterol, and triglycerides (TG) in fasting venous blood plasma. Tests were conducted at the Central Laboratory, SPSK2 in Szczecin. Measured glucose and insulin levels (fasting) were used to calculate two insulin resistance indices, HOMA and QUICKI calculated as (fasting insulin μM/mL) + (fasting glucose mg/dL)/405 (HOMAR) and 1/(log (fasting insulin μU/mL) + log (fasting glucose mg/dL) (QUICKI) [17]. All parameters were measured again after 10 treatments and 6 months after the last treatment. The determination of WBC, CRP, and the full lipid profile was also repeated 4 h after the end of the first treatment. We also analysed the plasma level of selected free fatty acids (FFAs) in fasting blood because the fact that the carbon chain length of most FAs in adipose tissue is between 10 and 22 atoms and contains 0 to 6 double bonds, including the most common fatty acids: oleic (C18: 1 n9), palmitic (C16: 1 n9) and linoleic (C 18: 2 n6) [18]. The level of FFAs was measured before the first treatment and again one and 4 h after the end of the first treatment, then immediately after 10 treatments, and 6 months after the completion of the treatment protocol. Fatty acids were extracted from 0.5 mL of serum using a modified Folch method, saponified with 2M KOH solution, and re-esterified with BF3 in methanol. Fatty acid methylene esters were analysed by gas chromatography (Agilent Technologies 7890A GC System, Agilent, US) on a 15 m × 0.10 mm, 0.10 μm capillary column (Supelcowax™ 10 Capillary GC Column, Supelco, Bellefonte, PA, USA). The parameters for the chromatographic separation were as follows: initial temperature 60 °C (0 min), then increased by 40 °C/min to 160 °C (0 min), with subsequent temperature increases of 30 °C/min to 190 °C (0.5 min). In the next step, the increase was also 30 °C/min and continued until 230 °C was reached (2.6 min). The temperature of 230 °C was maintained for 4.6 min. The entire analysis took about 8 min. The flow rate of carrier gas (hydrogen) was 0.8 mL/min [19]. 

### 2.4. Statistical Analysis

All continuous variables are expressed as a median and interquartile range [Q1–Q3] as not normally distributed. The Shapiro–Wilk test was used to check if a continuous variable *follows a normal distribution.* *Intergroup comparisons were performed with the Friedman repeated measures* analysis of variance by ranks and Kendall’s coefficient of concordance, Wilcoxon test, or Kruskal–Wallis one-way analysis of variance by ranks. Differences were statistically significant at *p* < 0.05. Statistics were processed with Statistica software, version 13.3 (StatSoft, Krakow, Poland).

## 3. Results

The baseline clinical, anthropometric, and laboratory characteristics of the study population, along with the short and long-term effects of all interventions applied for adipose tissue reduction, are presented in Table 1. A series of 10 treatments significantly reduced body weight, BMI, and waist circumference, and the effects persisted for 6 months after treatments. The performed treatments had no effect on the other analysed parameters. The direct effect of the first treatment assessed 4 h after the procedure (Table 2) showed an increase in inflammatory parameters (increased WBC count) but did not influence the level of lipids. 

There were no significant differences between baseline anthropometric and biochemical measurements in the U, RF, and RF/U subgroups. When considering the effect of individual interventions to reduce adipose tissue on the analysed parameters, significant differences were found between treatments. However, they were limited to body weight, waist circumference, and BMI. All treatments resulted in a reduction in body weight and BMI after 10 treatments, but the long-term effect evaluated 6 months after the last treatment was only found in the RF subgroup. None of the methods used alone resulted in short- and/or long-term hip circumference reduction. Short-term reduction in waist circumference was found for the RF and RF/U interventions, but not for U. Long-term reduction in waist circumference assessed 6 months after treatment was only achieved for the RF intervention (Table 3, Table 4, Table 5 and Table 6). 

Blood tests measuring the level of FAs showed no direct effect of the treatments on the early and short-term concentrations of the analysed FAs, regardless of the treatment applied. With a single exception (C:12 fatty acid analysed 4 h after RF treatment), there was no change in the concentration of free FAs one and 4 h after the procedure, nor after 10 treatments. RF and U treatments, but not RF/U, had a significant long-term effect on the levels of some FAs measured 6 months after the completion of treatments. In the RF subgroup, increased levels of C:15, C:18, C18:3*n*-6, and decreased levels of C10:0 were found. In the U subgroup, reduced levels of C:16, C18:1w9ct, C18:1trans1, C18:2n6c, and C20:4n6 were noted (Table 5). 

### Summary of Results

1. A series of 10 treatments to reduce abdominal adipose tissue using ultrasound, radiofrequency, or both methods resulted in a cosmetic effect reflected in weight loss and BMI reduction. Reduction in waist circumference was also found in patients who received radiofrequency or two types of intervention (RF + U), but not ultrasound treatments.
2. A long-term cosmetic effect (lasting for at least 6 months) was achieved only with RF treatment and was reflected in reduced body weight, BMI, and waist circumference.
3. None of the treatments had a direct, short-, or long-term effect on the lipid profile, insulin resistance markers, inflammation markers, or blood pressure.

## 4. Discussion

Adipose tissue is a unique tissue adapted to lipid storage and involved in immune, nervous and endocrine functions [4]. Adipose tissue acts as a buffer to fatty acids (FAs), just as the liver plays a buffer role in glucose homeostasis. FAs are stored and released from adipose tissue under hormonal factors and glucose availability [4]. Human adipose tissue is rich in lipid droplets with a phospholipids monolayer [20]. Each lipid droplet contains triglycerides (TG) with a wide range of FAs. The carbon chain in FAs mostly ranges from 10 to 22 atoms and 0 to 6 double bounds. Despite the variety of FAs, the proportions of monounsaturated (MUFA) and saturated compounds are relatively constant. The most abundant FA is oleic acid (C18:1 n9), which accounts for 40 to 50% of all FAs present in human tissue. Palmitic acid (C16:1 n9), the second most common MUFA, ranges from 5 to 7% of FAs. Linoleic acid (C 18:2 n6) accounts for approximately 2.6% of all FAs and has the largest variations among FAs [18]. Adipose tissue stores FAs as a part of TG, while serum fatty acids can be stored as part of TG or free FAs [4]. Even though the profile of serum FAs is reflected by FAs delivered from the diet, studies indicate that the composition of FAs in the blood can correlate with FA composition in adipose tissue. This observation concerns mostly groups of FAs, especially polyunsaturated fatty acids (PUFAs) [21]. 

Determination of fatty acid composition in adipose tissue and blood has been recognised as the gold standard for evaluating the quality of FAs consumed by individuals who do not experience sudden changes in their body weight. It was reported that the fatty acid composition of adipose tissue reflects the dietary intake of FAs in the previous 6 to 9 months [3]. Additionally, some studies [22,23] have indicated a difference in the fatty acid composition depending on the location of adipocytes. Subcutaneous adipose tissue in the abdominal region contains more saturated FAs and less monounsaturated FAs compared to adipocytes located in the buttock area. Interestingly, subcutaneous adipose tissue contains more monounsaturated FAs (but less saturated FAs) than visceral adipose tissue [23].

Excessive body weight is becoming one of the major global health care problems. It contributes to increased morbidity and mortality and generates high costs of health care [20]. Therefore, it is reasonable to emphasise the importance of a rational diet, as well as regular, moderate-intensity physical activity. The management of patients with metabolic syndrome should focus on lifestyle modification, which is the most effective method [24]. Non-invasive treatments offered by cosmetology or aesthetic medicine aimed at body contouring might provide additional interesting support in weight loss [18]. 

Adipose tissue in a healthy woman should account for 20–25% of body mass. Obesity in adults is diagnosed when the content of fat in the body, regardless of age, exceeds 25% of normal body weight in men and 30% in women. The content of adipose tissue is 14–28% in women with normal body weight, 29–32% in overweight women, and more than 32% in those with obesity. The state of overweight and obesity is determined by the Body Mass Index (BMI), calculated as the quotient of body weight (in kilograms) and the square of height expressed in meters. For the adult population, normal BMI is in the range of 18.5–24.9, 25–29.9 kg/m^2^ indicates overweight, and values higher than 30 kg/m^2^ indicate obesity. However, BMI does not provide information on the location of adipose tissue in the body [25]. 

The observed effect on the profile of FAs in patients’ blood is interesting. It is known that an increased level of FFAs in the blood serum is noted in obese and diabetic patients. This status is correlated with moderate chronic inflammation, which increases the risk of developing fatty liver disease and cardiovascular diseases [21]. Although the BMI of the analysed women indicated they were only slightly overweight (BMI 25.8 kg/m^2^), the waist circumference (81.9 cm) showed a tendency to accumulate subcutaneous abdominal fat. In addition, the mean HOMA-IR index was 2.2 (interpreted as the presence of insulin resistance [26]) and suggested that the studied women were at risk of developing components of metabolic syndrome. Change in lifestyle, including normalisation of body weight through regular moderate-vigorous physical activity and a balanced weight loss diet with a low glycemic index, rich in antioxidants, as well as mono- and PUFAs, with a limited intake of saturated fatty acids <10% and maximum reduction of trans-FAs (e.g., Mediterranean diet), is crucial in the treatment of metabolic syndrome [27].

It seems that the women who received treatments in our study began to pay attention to their diet, which was confirmed by the changes in the content of individual FAs in the blood. Our findings suggest that especially women who received ultrasound treatments reduced their daily fat intake, which was reflected in a significantly reduced concentration of the following FAs in the blood 6 months after the first treatment: C10:0 (14.2 vs. 5.4), C14:0 (27.3 vs. 14.5), C18:1w9ct (393 vs. 155.8), C18:1trans11 (55.3 vs. 34.9), C18:2n6c (523 vs. 195.4), C20:4n6 (147.8 vs. 53). Similar changes in the RF group were found only for C12: 0 (124.1 vs. 64.9). In the RF group, there was a significant decrease in the content of stearic acid in the blood after the last treatment (143.7 vs. 126.4), as well as an increase in its content after 6 months to the baseline value (143.7 vs. 204.3). This suggests that once patients achieved a satisfactory outcome after 10 treatments (reduction of the waist circumference from 82.4 to 80.9 cm and body weight from 73.2 to 72.4 kg), they liberalised their approach to a healthy diet. Consequently, six months after the completion of the study, the waist circumference of women from the RF group increased to 81.1 cm. That is why it is so important for specialists who offer cosmetology treatments to make their patients aware that maintaining reduced body weight and reducing the risk of developing the components of metabolic syndrome is possible only through a permanent change of diet. Cosmetological treatments can only serve as an addition to a healthy lifestyle, improve skin firmness, and sculpt the problematic parts of the body [28].

The content of adipose tissue can be precisely measured, for example, by densitometry or magnetic resonance [29,30,31]. However, these techniques for estimating body fat content require expensive specialist equipment and are not always available. The Bioelectrical Impedance Analysis (BIA) used in the present study is a noteworthy alternative. It is a non-invasive method of measuring body composition using tissues’ electrical properties. The analysis relies on measuring the electrical resistance consisting of the resistance and reactance of tissues that are penetrated by low intensity ≤ 1 mA and a frequency of 50 kHz current. In this study, a device with four electrodes placed on the abdomen was used [31]. Resistance is related to the specific resistance of tissues, while reactance is mainly related to the electrical capacity of cell membranes, which act as condensers. Tissues with a high content of water and electrolytes are characterised by good electrical conductivity, while adipose tissue has low water content and, therefore, low conductivity. Adipose tissue and extracellular water show active resistance [30].

In our study, we focused on two treatments, i.e., lipocavitation and thermolipolysis, which are used for body contouring, so they can mainly affect subcutaneous fat content in a selected body area.

In cosmetology, ultrasound is used, for example, for body sculpting and fat reduction treatments known as non-invasive liposuction [4,5,6,7,8,9,10]. Ultrasound is also used in aesthetic medicine, for example, for ultrasonic liposuction. However, these treatments use more energy and therefore must be performed by specialist doctors. 

Ultrasounds can ablate the adipose tissue by its nonthermal and thermal effects. Nonthermal-focused ultrasound applied for body sculpting uses mechanical stress generated from inertial cavitation to disrupt adipose tissue [9,11]. The thermal effect is characteristic mainly of high-frequency ultrasound (HIFU). In the thermal mechanism, heat generated by ultrasounds increases the focal spot temperature to above 58 °C, the upper limit of protein denaturation, leading to coagulative necrosis. The necrotic cells induce local inflammation, and the cellular debris and lipids from destroyed adipocytes are resorbed. Moreover, the wound healing cascade results in new collagen formation. These processes, proven in histopathological findings, result in skin tightening and reduction of fat volume [5,6,7]. Ultrasound may be appropriate for nonobese patients (body mass index < 30) with focal adiposity. Unfocused ultrasound with a frequency of 1 MHz (used in this study) is less absorbed by adipose tissue. Therefore, it induces a less pronounced thermal effect, unlike focused ultrasound with a frequency of 1–10 MHz and higher intensity, which is more strongly absorbed by adipose tissue. Ultrasound with a frequency of 1 MHz causes mechanical vibrations, increases the temperature of tissues, improves circulation and metabolism of the subcutaneous tissue, and also increases the permeability of the cell membrane in adipocytes [13,28].

According to some researchers, ultrasound induces lipocavitation with a frequency of 1 MHz and an intensity of 2 W/cm^2^. Lipocavitation can also be stimulated by intensities only slightly higher than 1 W/cm^2^ but with an adequately longer exposure time. This procedure damages adipocytes and causes the release of fat into the extracellular compartment. It is presumed that triglycerides released in this way are transported via vessels of the lymphatic system to the liver and are metabolised further [8,9,11,32].

Treatments with ultrasound alone had a less pronounced effect on reducing the values of the analysed parameters compared to the other two modalities. In our study, it might be related to unfocused ultrasound with a frequency of 1 MHz and intensity of 1.4 W/cm^2^. It can be assumed that the unfocused ultrasound used in the present study had a weaker effect on the lipocavitation process, which could have resulted in a slight reduction in the values of the analysed parameters. This is in agreement with the results of other authors [8].

On the other hand, electromagnetic (radiofrequency) waves with a frequency of 1 MHz and a power of 100 W (bipolar method) used in this study showed a stronger effect on adipose tissue compared to that produced by ultrasound. Endogenous heat is produced in tissues exposed to an electromagnetic field. The local increase in skin temperature results in the destruction of some cross-links between collagen fibres. Collagen is partially denatured, but more thermostable chemical bonds are unaffected. Collagen fibres shorten, which is clinically manifested by improved skin firmness [8,9,11]. Partially denatured collagen is degraded, which stimulates fibroblasts to synthesise new collagen and initiates the “remodelling” process, which can last from several weeks to several months. The heat generated during the treatment also enhances tissue metabolism in the treated area, reducing adipose tissue content. The described process could have taken place as a result of the radiofrequency treatments applied in our study and contributed to the reduction of the values of the analysed parameters, especially 30 days after the completion of 10 treatments. There are no standard protocols; in order to obtain desired results, several sessions are necessary, with the frequency between 3 kHz and 24 GHz on monopolar or bipolar devices [8,9,33]. 

One study subgroup received combined radiofrequency and ultrasound treatments. First, massage was performed with a bipolar applicator head that generates electromagnetic (radiofrequency) waves. The heat generation in the tissue exposed to radiofrequency waves probably created optimal conditions for ultrasound waves by enhancing their effect. It could also have had a more favourable effect on lowering the values of the analysed parameters compared to one type of treatment alone. Radiofrequency waves cause thermolipolysis and boost metabolism in adipocytes [8,9]. On the other hand, ultrasound improves the function of the lymphatic system, which indirectly reduces the amount of adipose tissue. 

Our study evaluating the effects of electromagnetic (radiofrequency) waves showed their significant impact on the body weight and BMI reduction of the studied women, contrary to some previously reported findings [4].

In some clinical trials, 6–10 radiofrequency treatments have produced 2- to 3.5 cm reductions in waist and thigh circumference. However, newer devices are more effective; in one study, a reduction in thigh circumference of 2.43 cm after only four weekly treatments was observed [34]. HIFU and radiofrequency are considered to be relatively safe treatment options, although some adverse events may occur. The most common include pain during or after the procedure, oedema, ecchymosis, dysesthesia, and erythema. These adverse events typically resolve within a couple of hours, but more serious complications (bradypnea, rash, herpes zoster) may require some interventions [5,6]. Since cosmetologists cannot prescribe any medications, they are limited to only over-the-counter medications. For newer methods, contactless radiofrequency applicators, no adverse effects were observed in a forty-subject study [34]. This may lead to the conclusion that novel radiofrequency applicators can be safely used in cosmetologists’ practice. 

The non-invasive physical methods used in our study can be applied primarily to prevent the excessive deposition of adipose tissue, as well as to support the treatment of overweight and obesity. However, they cannot be the basic method for reducing the volume of deposited adipose tissue, regardless of its location. Importantly, the combination of radiofrequency and ultrasound affected adipose tissue, resulting in a partial reduction of fat content, as well as improved skin elasticity. Other authors also revealed that some of the non-invasive body contouring devices in animal and human studies, such as cryolipolysis, RF, and HIFU, showed statistically significant effects on body contouring, removing unwanted fat in some body areas. However, the clinical effects are mild to moderate, for example, 2–4 cm circumference reduction as a sign of subcutaneous fat reduction during total treatment sessions [8]. 

Importantly, all the treatments mentioned in this study, because of the use of lower energy levels, can be performed by cosmetologists. The legal aspects of procedures performed by cosmetologists, ultrasound and radiofrequency treatments included, are at present not regulated in local legislation. Theoretical and practical training on these treatments and an evaluation of risk/benefits for each patient are necessary to perform these procedures effectively and safely.

An important informational and educational issue, which should be considered in beauty salons offering non-invasive physical treatments, is making their patients aware that long-term reduction of adipose tissue in individuals with overweight or obesity requires a change in lifestyle, including an appropriate diet and modification of energy expenditure by physical activity [32]. Evidence has shown that at least 150–300 min/week of physical activity of moderate intensity weekly or 75–150 min/week of vigorous physical activity is needed to prevent weight maintenance. However, an individual’s intent on losing weight should perform at least 200–300 min/week of moderate-to-vigorous physical activity [32].

## 5. Study Limitations

The study design did not include a control group. It is likely that the inclusion of individuals who did not undergo any abdominal adipose tissue intervention might have elucidated whether observed blood FAs changes were due to the treatment or caused by other interventions such as changes in dietary habits.

## 6. Conclusions

1. Treatments to reduce abdominal adipose tissue with ultrasound, radiofrequency, or both techniques provided a desired cosmetic effect. RF treatment was the most efficient modality since it produced a long-term cosmetic effect (lasting for at least 6 months), reflected in reduced body weight, BMI, and waist circumference. However, many biochemical cardiovascular risk factors remained intact. In fact, it is too early to determine whether a total cardiovascular risk has been modified due to a relatively short follow-up.
2. The techniques used in our study did not increase the content of pro-inflammatory free fatty acids in the blood (such as arachidonic acid), which suggests the safety of these treatments.

## Figures and Tables

**Table 1:** The clinical, anthropometric and biochemical characteristics of the study population and the short and long-term effects of all interventions applied for an adipose tissue reduction.

Parameters	Baseline	After 10 Treatments vs. Baseline	*p*	After 6 Months vs. Baseline	*p*
Age, years	38 (33–42)	-	-	-	-
Height, cm	167 (163–170)	-	-	-	-
Body weight, kg	68 (63–75.3)	67.3 (62–75.5)	0.001	66.7 (59–74.1)	0.005
BMI, kg/m^2^	25 (22.5–28.3)	24.7 (22.3–28)	0.001	24.7 (22.4–27.9)	0.005
Waist circumference, cm	82 (75–88)	78 (74–85)	0.001	78 (74–84)	0.02
Hip circumference, cm	101 (96–105)	100 (95–104)	0.5	98 (94–103)	0.3
WHR	0.81 (0.77–0.84)	0.81 (0.77–0.83)	0.9	0.81 (0.77–0.83)	0.9
HOMA	2.0 (1.2–2.7)	1.6 (1.2–2.5)	0.1	1.9 (1.2–2.5)	0.5
QUICKI	0.3 (0.3–0.4)	0.3 (0.3–0.4)	0.7	0.3 (0.3–0.4)	0.7
WBC, × 10^9^/L	6.1 (4.8–7.3)	6.1 (5.1–7.0)	0.8	6.1 (5.1–7.5)	0.9
CRP, mg/L	1.1 (0.5–2.0)	1.1 (0.4–2.0)	0.8	1.1 (0.5–2.1)	0.8
Glucose, mg/dL	92 (86–99)	91 (85–96)	0.6	90 (84–95)	0.4
Insulin, I.U.	8.6 (5.5–11.5)	7.1 (5.5–12.5)	0.2	8.8 (5.4–11.4)	0.6
Total cholesterol, mg/dL	197 (175–217)	191 (172–215)	0.3	196 (165–215)	0.8
LDL, mg/dL	109 (90–127)	106 (94–127)	0.5	110 (91–132)	0.9
HDL, mg/dL	59 (50–67)	56 (50–65)	0.6	60 (50–65)	0.7
TG, mg/dL	76 (59–105)	74 (58–111)	0.7	83 (62–109)	0.5
Systolic pressure, mmHg	110 (110–120)	115 (105–120)	0.2	115 (105–130)	0.4
Diastolic pressure, mmHg	70 (60–80)	70 (60–75)	0.9	70 (65–80)	0.9

Abbreviations: BMI, body mass index; WHR, waist to hip ratio; WBC, white blood cell; CRP, C-reactive protein; LDL, cholesterol; HDL, cholesterol plus triglycerides; TG, triglycerides; HOMA, homeostatic model assessment; QUICKI, quantitative insulin sensitivity check index; Differences were statistically significant at *p* < 0.05. All continuous variables are expressed as a median and interquartile range [Q1–Q3] as not normally distributed. The Shapiro–Wilk test was used to check if a continuous variable follows a normal distribution.

**Table 2:** Inflammatory and biochemical parameters in the study population.

Parameters	Baseline	4 h After Treatment	*p*
WBC, ×10^9^/L	6.1 (4.8–7.3)	7.3 (5.8–8.4)	0.005
CRP, mg/L	1.1 (0.5–2.0)	1.1 (0.5–2.0)	0.7
TC, mg/dL	197 (175–217)	196 (170–222)	0.8
HDL, mg/dL	59 (50–67)	60 (50–72)	0.4
LDL, mg/dL	109 (90–127)	107 (89–131)	0.8
TG, mg/dL	76 (59–105)	73 (57–93)	0.5

Abbreviations are the same as under Table 1. All continuous variables are expressed as a median and interquartile range [Q1–Q3] as not normally distributed. The Shapiro–Wilk test was used to check if a continuous variable follows a normal distribution. Differences were statistically significant at *p* < 0.05.

**Table 3:** Body weight of the studied women [kg].

Treatment	Baseline	After 10 Treatments vs. Baseline	*p*	After 6 Months vs. Baseline	*p*
U	63.3 (60–70)	62.9 (58.8–68.7)	0.01	62.5 (58–72.6)	0.1
RF	69.75 (64–79.2)	67.6 (63.5–78)	0.0035	67.75 (63.8–77)	0.04
RF/U	68 (64.5–82.8)	67.75 (64.3–81.5)	0.009	68.1 (62.5–80)	0.3

Abbreviations: Destination U, group treated with ultrasound; RF, group treated using radiofrequency device. All continuous variables are expressed as a median and interquartile range [Q1–Q3] as not normally distributed. The Shapiro–Wilk test was used to check if a continuous variable follows a normal distribution. Differences were statistically significant at *p* < 0.05.

**Table 4:** BMI in the studied women [kg/m^2^].

Treatment	Baseline	After 10 Treatments vs. Baseline	*p*	After 6 Months vs. Baseline	*p*
U	23.7 (22.3–25.6)	23.3 (22.2–25.3)	0.02	23.1 (22.1–25.5)	0.2
RF	25.7 (23.3–30.4)	25.2 (23.2–30)	0.002	25.1 (23.2–30)	0.03
RF/U	26.1 (22–28.6)	25.9 (22–28.7)	0.003	25.3 (21.6–29.8)	0.4

Designation is the same as under Table 3. All continuous variables are expressed as a median and interquartile range [Q1–Q3] as not normally distributed. The Shapiro–Wilk test was used to check if a continuous variable follows a normal distribution. Differences were statistically significant at *p* < 0.05.

**Table 5:** Waist circumference of the studied women [cm].

Treatment	Baseline	After 10 Treatments vs. Baseline	*p*	After 6 Months vs. Baseline	*p*
U	78.5 (75–88)	78 (74–85)	NS	78 (73–84)	0.9
RF	82.5 (77–92)	78.5 (75–86)	0.008	78.5 (76–83)	0.04
RF/U	82 (76–85)	77 (75–83)	0.02	76 (74–83)	0.5

Designation is the same as under Table 3. All continuous variables are expressed as a median and interquartile range [Q1–Q3] as not normally distributed. The Shapiro–Wilk test was used to check if a continuous variable follows a normal distribution. Differences were statistically significant at *p* < 0.05.

**Table 6:** Content of fatty acids in the blood [ug/mL].

Time Point Treatment	FFAs [ug/mL]	Before	1 h After	*p* vs. Before	4 h After	*p* vs. Before	After the Last Treatment	*p* vs. Before	6 Months After	*p* vs. Before
U	C10:0	9 (7–15)	9 (9–15)	0.8	12 (7–16)	0.09	14 (8–21)	0.07	4 (2–9)	0.06
RF		19 (12–27)	16 (20–22)	0.5	18 (12–25)	0.7	23 (16–27)	0.5	5 (3–9)	0.013
RF/U		8 (5–12)	9 (7–16)	0.9	9 (5–15)	0.8	12 (8–16)	0.6	10 (9–12)	0.7
U	C12:0	4 (2–21)	4 (2–59)	0.6	12 (2–164)	0.1	5 (3–263)	0.5	5 (2–76)	0.7
RF		27 (7–257)	57(6–231)	0.2	105 (12–288)	0.03	31(15–277)	0.09	63(10–120)	0.2
RF/U		3 (2–9)	4 (2–17)	0.7	4 (3–8)	0.8	3 (3–10)	0.7	3 (2–10)	0.6
U	C14:0	8 (5–12)	9 (7–16)	0.6	9 (5–15)	0.8	12 (8–16)	0.7	10 (9–12)	0.8
RF		10 (9–15)	10 (9–13)	0.7	10 (9–13)	0.7	10(8–17)	0.6	22 (12–23)	0.3
RF/U		17 (14–24)	16 (10–19)	0.9	15 (11–18)	0.9	23 (17–26)	0.5	17 (10–20)	0.9
U	C15:0	4 (3–4)	3 (3–4)	0.4	3 (2–4)	0.6	4 (3–17)	0.7	3 (2–5)	0.6
RF		2 (2–3)	2 (2–3)	0.8	2 (2–4)	0.06	3 (2–3)	0.1	5 (4–6)	0.005
RF/U		3 (2–4)	2 (2–4)	0.6	4 (2–5)	0.3	4 (2–5)	0.4	2 (2–4)	0.6
U	C16:0	345 (275–474)	352 (250–408)	0.4	346 (302–390)	0.6	414 (351–433)	0.09	292 (241–353)	0.03
RF		300 (243–350)	320 (244–361)	0.7	302 (236–318)	0.7	283 (258–294)	0.5	405 (278–445)	0.2
RF/U		384 (272–498)	307 (277–411)	0.8	424 (250–578)	0.8	465 (344–587)	0.5	382 (300–404)	0.9
U	C16:1	22 (15–28)	18 (8–27)	0.6	22 (19–27)	0.8	32 (20–40)	0.1	15 (13–18)	0.3
RF		15 (10–18)	17 (14–35)	0.8	19 (16–26)	0.7	15(14–23)	0.7	17 (12–21)	0.8
RF/U		22 (15–36)	19 (12–32)	0.8	14 (13–33)	0.4	35 (18–44)	0.5	22 (14–31)	0.9
U	C17:0	6 (4–9)	5 (4–9)	0.5	4 (4–5)	0.05	6 (4–8)	0.6	4 (3–14)	0.3
RF		5 (4–9)	5 (4–6)	0.6	5 (4–6)	0.7	5(4–5)	0.8	11 (5–16)	0.07
RF/U		5 (4–7)	4 (3–8)	0.7	6 (3–8)	0.6	6 (4–8)	0.7	4 (3–6)	0.7
U	C18:0	148 (108–178)	139 (103–153)	0.8	147 (115–160)	0.8	149 (143–245)	0.8	132 (112–238)	0.5
RF		130 (109–144)	115 (97–141)	0.7	127 (98–131)	0.5	121(115–131)	0.7	224 (144–270)	0.046
RF/U		172 (134–202)	153 (123–198)	0.3	173 (113–244)	0.8	203 (153–234)	0.4	153 (126–175)	0.8
U	C18:1w9ct	271 (162–363)	223 (167–267)	0.8	232 (205–269)	0.5	258 (215–348)	0.8	142 (104–185)	0.01
RF		224 (165–252)	207 (146–293)	0.7	220(169–256)	0.7	205(179–272)	0.6	168 (138–218)	0.1
RF/U		285 (169–373)	262 (176–315)	0.7	324 (172–415)	0.3	331 (237–423)	0.5	272 (180–320)	0.9
U	C18:1trans1	28 (23–41)	23 (16–29)	0.4	21 (21–26)	0.6	28 (24–35)	0.9	17 (13–23)	0.03
RF		20 (15–27)	21(15–31)	0.7	20 (17–25)	0.7	18(17–26)	0.7	16 (16–17)	0.3
RF/U		28 (13–35)	25 (15–28)	0.8	30 (15–42)	0.7	33 (21–36)	0.7	24 (17–29)	0.6
U	C18:2n6c	281 (232–360)	263 (200–349)	0.6	278 (236–308)	0.7	293 (230–401)	0.6	202 (137–244)	0.02
RF		299 (219–377)	293 (236–361)	0.8	296 (242–327)	0.7	316(269–331)	0.4	226 (201–279)	0.08
RF/U		348 (212–387)	273 (192–343)	0.5	306 (204–476)	0.6	361 (308–461)	0.7	284 (236–314)	0.2
U	C18:3*n*-6	4 (4–5)	4 (2–4)	0.7	3 (3–5)	0.6	4 (3–7)	0.7	3 (2–11)	0.7
RF		4 (3–5)	4 (3–4)	0.8	4 (4–5)	0.08	4(3–5)	0.8	5 (4–10)	0.03
RF/U		4 (3–5)	4 (3–5)	0.7	4 (3–7)	0.3	4 (3–6)	0.4	4 (3–5)	0.8
U	C20:4n6	84 (75–98)	82 (67–91)	0.9	84 (67–91)	0.9	102 (81–138)	0.3	47 (36–72)	0.07
RF		67 (51–83)	72 (57–94)	0.9	76 (62–85)	0.8	77 (62–85)	0.8	59 (52–63)	0.5
RF/U		84 (52–110)	64 (55–88)	0.1	85 (43–138)	0.7	89 (77–122)	0.8	70 (65–75)	0.4

Designation is the same as under Table 3. All continuous variables are expressed as a median and interquartile range [Q1–Q3] as not normally distributed. The Shapiro–Wilk test was used to check if a continuous variable follows a normal distribution. Differences were statistically significant at *p* < 0.05.

## Data Availability

Not applicable.
